# A retrospective study of the effects of a vasopressor bolus on systolic slope (dP/dt) and dynamic arterial elastance (Ea_dyn_)

**DOI:** 10.1186/s12871-024-02574-x

**Published:** 2024-07-29

**Authors:** Alexa C. Abdallah, Sang H Song, Neal W. Fleming

**Affiliations:** 1 Department of Anesthesiology, University of California, San Diego, CA USA; 2 Sutter Medical Center, Sacramento, CA USA; 3 Department of Anesthesiology & Pain Medicine, University of California, Davis, 4150 V Street Suite 1200 PSSB, Sacramento, CA 95817 USA

**Keywords:** Systolic slope (dP/dt), Dynamic arterial elastance (Ea_dyn_), Hypotension prediction index (HPI), Phenylephrine, Ephedrine, Vasoconstrictor

## Abstract

**Background:**

To enhance the utility of functional hemodynamic monitoring, the variables systolic slope (dP/dt) and dynamic arterial elastance (Ea_dyn_) are calculated by the Hypotension Prediction Index (HPI) Acumen® Software. This study was designed to characterize the effects of phenylephrine and ephedrine on dP/dt and Ea_dyn_.

**Methods:**

This was a retrospective, non-randomized analysis of data collected during two clinical studies. All patients required intra-operative controlled mechanical ventilation and had an indwelling radial artery catheter connected to an Acumen IQ sensor. Raw arterial pressure waveform data was downloaded from the patient monitor and all hemodynamic measurements were calculated off-line. The anesthetic record was reviewed for bolus administrations of either phenylephrine or ephedrine. Cardiovascular variables prior to drug administration were compared to those following vasopressor administrations. The primary outcome was the difference for dP/dt and Ea_dyn_ at baseline compared with the average after the bolus administration. All data sets demonstrated non-normal distributions so statistical analysis of paired and unpaired data followed the Wilcoxon matched pairs signed-rank test or Mann-Whitney U test, respectively.

**Results:**

201 doses of phenylephrine and 100 doses of ephedrine were analyzed. All data sets are reported as median [95% CI]. Mean arterial pressure (MAP) increased from 62 [54,68] to 78 [76,80] mmHg following phenylephrine and from 59 [55,62] to 80 [77,83] mmHg following ephedrine. Stroke volume and cardiac output both increased. Stroke volume variation and pulse pressure variation decreased. Both drugs produced significant increases in dP/dt, from 571 [531, 645] to 767 [733, 811] mmHg/sec for phenylephrine and from 537 [509, 596] to 848 [779, 930] mmHg/sec for ephedrine. No significant changes in Ea_dyn_ were observed.

**Conclusion:**

Bolus administration of phenylephrine or ephedrine increases dP/dt but does not change Ea_dyn_. dP/dt demonstrates potential for predicting the inotropic response to phenylephrine or ephedrine, providing guidance for the most efficacious vasopressor when treating hypotension.

**Trial registration:**

Data was collected from two protocols. The first was deemed to not require written, informed consent by the Institutional Review Board (IRB). The second was IRB-approved (Effect of Diastolic Dysfunction on Dynamic Cardiac Monitors) and registered on ClinicalTrials.gov (NCT04177225).

## Background

Intraoperative hypotension is common in patients undergoing general anesthesia [1, 2]. Maintaining optimal intraoperative hemodynamics is paramount, as strong correlations exist between hypotension and the incidence of postoperative myocardial infarction, acute kidney injury, and mortality [3–5]. Hypotension can be broadly considered as pathology related to inadequate fluid resuscitation, vasomotor tone, and/or cardiac function. As such, the treatment of hypotension includes identifying the cause and administering intravenous fluids, vasoconstrictors, and/or inotropes, respectively [6]. Determining the most efficacious treatment for a hypotensive episode can be elusive as blood pressure is determined by complex interactions between cardiac stroke volume and arterial tone [7, 8]. 

The HemoSphere patient monitor with Hypotension Prediction Index (HPI) Acumen® software (Edwards Lifesciences, Irvine, CA) is designed to guide intraoperative therapy and minimize hypotension. Machine learning was used to develop an algorithm that can predict an impending hypotensive event based on the integration of selected arterial waveform characteristics [9]. The HPI software has been validated as a reliable predictive index in both cardiac [10] and non-cardiac [11] surgical procedures and has been shown to decrease the total duration of intraoperative hypotension [12]. This software provides hemodynamic variables that characterize contractility, (systolic slope, dP/dt) and vascular tone (dynamic arterial elastance, Ea_dyn_). These variables may provide therapeutic guidance for treatment of hypotension. Characterization of their response to commonly used vasoconstrictors may improve patient management. This study examined specifically how these variables respond to phenylephrine and ephedrine.

## Methods

Data collected from two protocols were included for this single center study. The first protocol was an evaluation of non-invasive cardiac output monitors in patients undergoing elective cardiac surgery. This study was deemed to not require written, informed consent by the Institutional Review Board (IRB) as part of a quality improvement project. The primary results from this evaluating the utility of the HPI in cardiac surgical patients has been published [10]. The second protocol was an IRB-approved prospective evaluation of the effects of diastolic function on the dynamic monitors of cardiac function (Effect of Diastolic Dysfunction on Dynamic Cardiac Monitors, NCT04177225, first registered 26/11/2019). The primary data from this protocol is still being evaluated.

All patients in this second protocol provided written, informed consent. All patients required controlled mechanical ventilation and a radial artery pressure monitoring catheter connected to Acumen IQ sensor and a physiological monitor (EV-1000 or HemoSphere) that contained the HPI software (Edwards Lifesciences LLC, Irvine, CA). Post-operatively, the raw waveform data was downloaded from the physiological monitor. All hemodynamic parameters were calculated off-line at twenty second intervals. The following parameters were extracted and evaluated: mean arterial pressure (MAP), heart rate (HR), stroke volume (SV), cardiac output (CO), stroke volume variation (SVV), pulse pressure variation (PPV), systolic slope (dP/dt), and dynamic arterial elastance (Ea_dyn_).

Each patient’s electronic medical record was reviewed for bolus administrations of either phenylephrine or ephedrine. Medications administered during cardiopulmonary bypass were excluded. Given the potential for imprecise charting for medication administration, drug administration time was defined as the time with the lowest blood pressure occurring within 3 min of the electronic medical record documented drug administration. Baseline cardiovascular variables were determined as the average over three minutes prior to drug administration. The primary outcome was the difference for the cardiovascular variables dP/dt and Ea_dyn_ at baseline compared with the average taken over three minutes beginning two minutes after the bolus vasopressor administration.

### Statistical analysis

Initial review of the scatter plots for each hemodynamic variable revealed apparent outliers. For each value associated with a change ≥ 3 standard deviations of the average values for that variable, the primary data were reviewed. If the primary data values appeared to be physiologically unlikely, probably reflecting a recording artifact, all variables associated with that drug administration were removed from the final analysis. For all measured hemodynamic variables, the distribution of each final data set was assessed using the D’Agostino Pearson normality test. All data sets demonstrated non-normal distributions. Statistical analysis of paired and unpaired data followed the Wilcoxon matched pairs signed-rank test or Mann-Whitney U test, respectively using GraphPad Prism version 10.2.1 for Windows, GraphPad Software, San Diego, California USA, www.graphpad.com. An alpha of 0.05 was applied to determine statistical significance.

## Results

Data from 101 patients undergoing elective, non-laparoscopic cardiac, vascular, intra-abdominal oncologic, or head and neck cancer surgery collected between January 2019 and April 2020 at the University of California, Davis Medical Center were included in this study. 72 were male, 29 were female with an average age of 69 ± 10.8 years, average weight of 82 ± 18.0 kg, and average height of 173 ± 9.3 cm. Four vasopressor doses were removed from analysis as outliers or artifacts. The MAP response for different doses of phenylephrine (50, 80, and 100 mcg) were not statistically different (12 ± 12, 22 ± 12, 18 ± 14 mmHg, respectively). The MAP response for different doses of ephedrine (5 and 10 mg), were not statistically different (23 ± 16, 23 ± 16 mmHg, respectively). Thus, all doses of phenylephrine were combined to comprise the data set for each hemodynamic parameter extracted; likewise, for all doses of ephedrine. A total of 201 doses of phenylephrine (doses/patient: mean 2.73, median 2, maximum 9) and 100 doses of ephedrine (doses/patient: mean 1.98, median 2, maximum 8) were included for analysis.

**Fig. 1:**
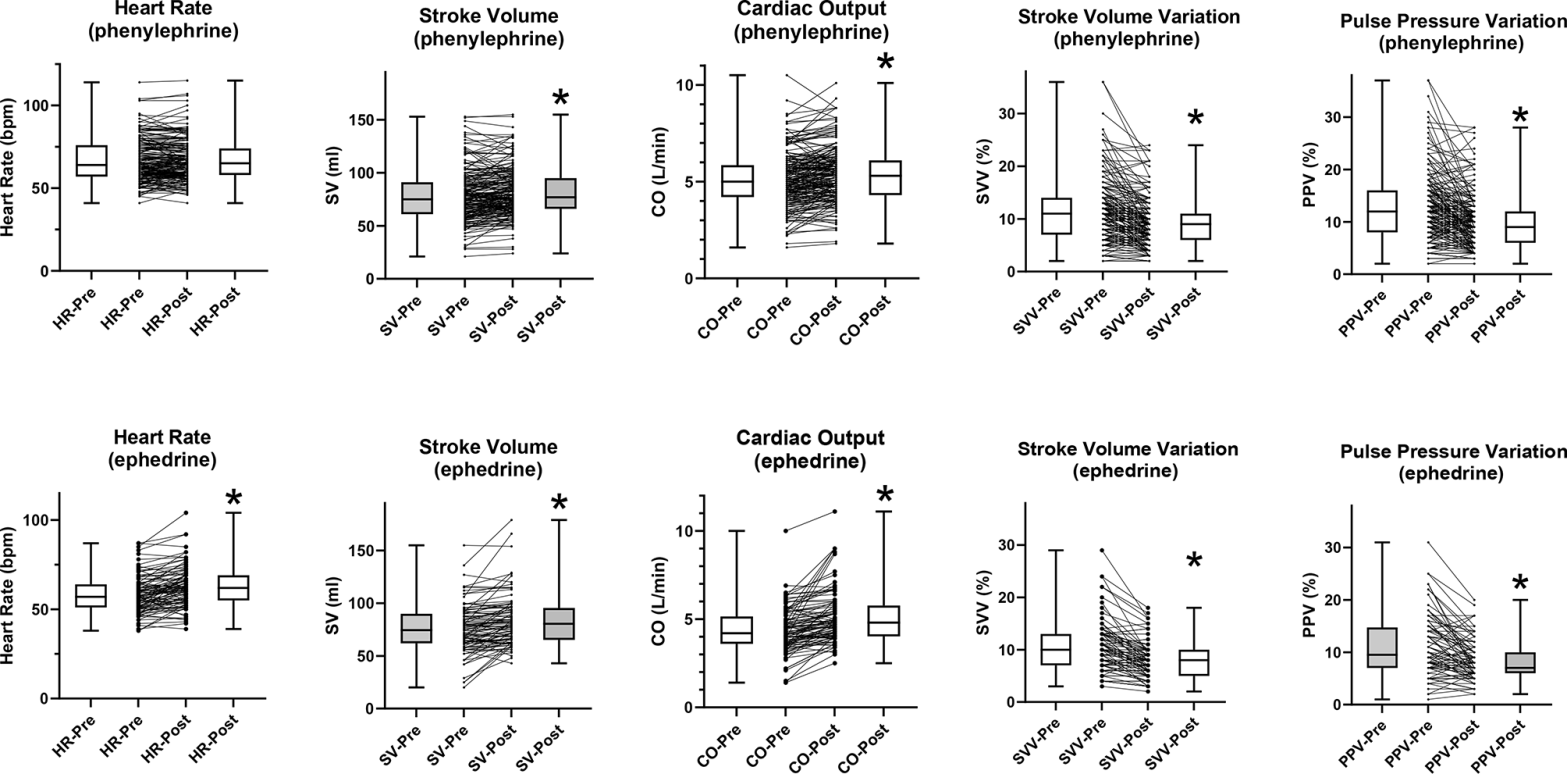
Hemodynamic responses to phenylephrine and ephedrine as absolute values. Paired comparisons to baseline values, “*” indicates *p* < 0.05

The changes in the absolute values of each hemodynamic variable are provided in Table 1. The bolus administration of phenylephrine was associated with an average increase of 28 ± 22% in MAP (median:23%, 95% CI:20.6–27.3). There was no significant change in heart rate (average 1 ± 8%, median 0.1%, 95% CI: -0.6–1.2). The stroke volume significantly increased by an average of 6 ± 15.7% (median: 3%, 95% CI: 1.8–4.6) with a consequent average increase in the cardiac output of 7 ± 16% (median:5%, 95%CI:3.5–6.1). The stroke volume variation significantly decreased by an average 14 ± 30% (median: -15%, 95% CI: -19.5 to -11.3) as did the pulse pressure variation by an average 12 ± 32.5% (median: -14%, 95% CI: -18.8 to -7.5). (Fig. 1)

**Table 1:** Hemodynamic Variables - Absolute Values

Phenylephrine	MAP (mmHg)		HR (beats/ min)		SV (ml)		CO (L/min)		SVV (%)		PPV (%)		dP/dt (mmHg/sec)		Ea_dyn_	
	Pre	Post	Pre	Post	Pre	Post	Pre	Post	Pre	Post	Pre	Post	Pre	Post	Pre	Post
**Median**	62	78	64	65	75	77	5	5.3	11	9	12	9	571	767	1.1	1.1
**95% CI**	61–64	76–80	62–66	63–67	72–80	74–82	4.7–5.2	5- 5.5	10–11	8–9	11–12	9–10	531–645	733–811	1.0–1.1	1.1–1.2
**Ephedrine**																
	Pre	Post	Pre	Post	Pre	Post	Pre	Post	Pre	Post	Pre	Post	Pre	Post	Pre	Post
**Median**	59	80	57	62	75	81	4.2	4.8	10	8	9.5	7	537	848	1.02	1.05
**95% CI**	55–62	77–83	55–60	60–65	68–80	73–84	4-4.6	4.6-5	9–11	7–8	8–11	6–8	509–596	779–930	0.97–1.06	0.97–1.12

Data are presented as median, 95% CI. Each hemodynamic parameter column indicates the combined analysis of absolute values of all vasopressor doses 3 min prior to drug administration (Pre), and the average two to five minutes following administration (Post)

*Abbreviations* MAP, mean arterial pressure; HR, heart rate; SV, stroke volume; CO, cardiac output; SVV, stroke volume variation; PPV, pulse pressure variation; dP/dt, maximum systolic slope; Ea_dyn_, dynamic arterial elastance

**Fig. 2:**
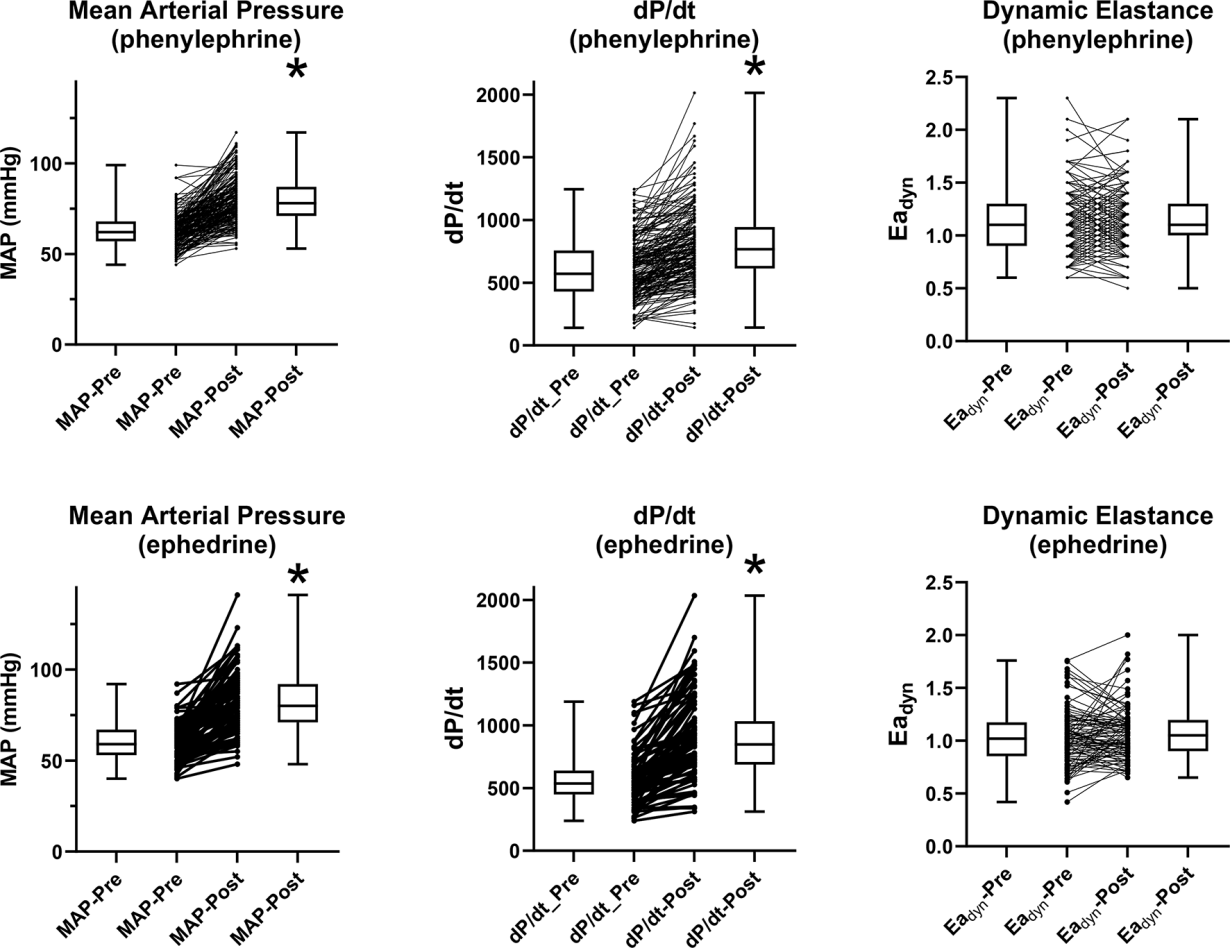
Hemodynamic variable responses to phenylephrine or ephedrine in absolute values. Paired comparisons to baseline values, “*” indicates *p* < 0.05

The systolic slope (dP/dt) increased significantly by 41 ± 51% (median:26%, 95% CI: 20.8–31.9) but there was no significant change in the dynamic arterial elastance (Ea_dyn_), average 4 ± 20% (median: 3%, 95% CI -0.4–5.8). (Fig. 2) For ephedrine, the bolus administration was associated with an average increase of 40 ± 30% in MAP (median:31%, 95% CI:26.2–43.6). Heart rate increased significantly by an average of 8 ± 12% (median:6%, 95% CI: 3.5–9.4) The stroke volume also increased significantly by an average of 14 ± 31.8% (median: 4%, 95% CI: 2.8–8.1), combining with the increased heart rate to produce an average increase of 21 ± 32% in the cardiac output (median:12%, 95% CI:7.8–16.5). The stroke volume variation significantly decreased by an average of 20 ± 24% (median: -22%, 95% CI: -26 to -15) with a similarly significant decrease of 15 ± 35.7% (median: -24%, 95% CI: -30.6 to -11.2) in the pulse pressure variation. (Fig. 1) The systolic slope (dP/dt) increased significantly by 63 ± 54% (median:52%, 95% CI: 36.5–66.1) but there was no significant change in the dynamic arterial elastance (Ea_dyn_), average 9 ± 36% (median: -1%, 95% CI -5.6–3.4). (Fig. 2)

**Fig. 3:**
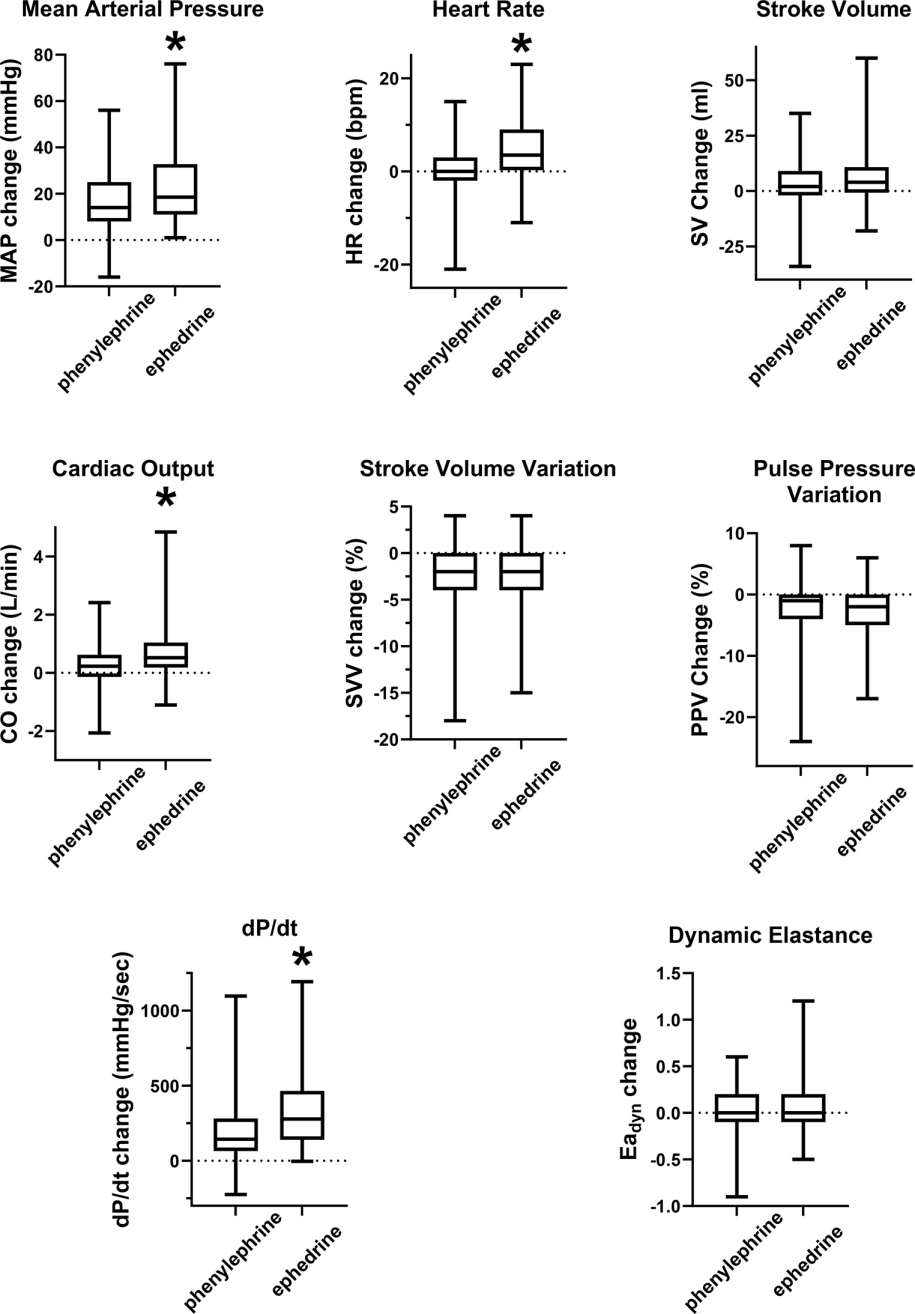
Hemodynamic variable response to phenylephrine or ephedrine, change in absolute values. Group comparisons between drugs, “*” indicates *p* < 0.05

When the responses to phenylephrine and ephedrine were compared, the average increase in MAP (17 ± 13 mmHg vs. 23 ± 16 mmHg) was comparable. However, ephedrine produced a significant increase in heart rate (0.5 ± 5 vs. 4 ± 6 beats per minute) and while the increases in stroke volume were comparable (3 ± 10 vs. 7 ± 13 ml), ephedrine produced a greater increase in cardiac output (0.2 ± 0.8 vs. 0.7 ± 1.0 L per minute). The effects on SVV and PPV were comparable for both drugs (-2 ± 3.8 vs. -3 ± 3.3% and − 2 ± 4.9 vs. -3 ± 4.3%, respectively). The increase in dP/dt (198 ± 202 vs. 330 ± 245 mmHg/sec) was greater for ephedrine in comparison to phenylephrine, while there was no difference in the changes for Ea_dyn_ (0.02 ± 0.23 vs. 0.04 ± 0.28) between the two drugs. (Fig. 3).

Secondary analysis was then performed to evaluate the influence of the baseline intravascular volume status, as characterized by the SVV, on the observed responses. Patients with an SVV ≥ 13% were considered to be volume responsive. Weak correlations were observed between the baseline SVV and the increase in MAP, SV and dP/dt following vasopressor administration. These correlations were slightly stronger following ephedrine. No correlations were observed for the changes in HR or Ea_dyn_. (Table 2) For within group comparisons following the administration of phenylephrine, only the change in dP/dt (*p* = 0.02) was greater in the volume responsive patients as compared to the non-volume responsive patients. Following ephedrine, the increases in CO (*p* = 0.0004), SV (*p* = 0.003), MAP (*p* = 0.003) and dP/dt (*p* < 0.0001) were all greater in the volume responsive patients. For comparisons between groups, in the non-volume responsive patients, the increases in CO (*p* = 0.001), HR (*p* < 0.0001) and dP/dt (*p* = 0.001) were all greater following administration of ephedrine. In the volume responsive patients, the increases in CO (*p* = 0.001), SV (*p* = 0.045), HR (*p* < 0.001), MAP (*p* = 0.002) and dP/dt (*p* = 0.0005) were also greater following the administration of ephedrine.

**Table 2:** Correlations Between SVV and Hemodynamic Variables

		Drug	
Variable	Pearson	Phenylephrine	Ephedrine
MAP	r	0.20	0.42
	95% CI	0.06, 0.33	0.24, 0.58
HR	r	-0.04	0.01
	95% CI	-0.18, 0.11	-0.20, 0.21
SV	r	0.16	0.33
	95% CI	0.02, 0.30	0.14, 0.50
dP/dt	r	0.22	0.38
	95% CI	0.08, 0.35	0.19, 0.54
Ea_dyn_	r	-0.07	-0.13
	95% CI	-0.21, 0.07	-0.32, 0.08

Correlations between SVV and each variable response to vasopressor administration were examined to evaluate the impact of preload on the observations

*Abbreviations* SVV, stroke volume variation; MAP, mean arterial pressure; HR, heart rate; SV, stroke volume; dP/dt, maximum systolic slope; Ea_dyn_, dynamic arterial elastance

## Discussion

The hemodynamic variables of maximal systolic slope (dP/dt) and dynamic arterial elastance (Ea_dyn_) are not new [13–15], however, with the HPI Acumen^®^ software, they are readily available to potentially guide intra-operative blood pressure management. dP/dt provides a correlate of myocardial contractility [16–18] while Ea_dyn_ provides a characterization of vascular resistance [7, 15]. Because clinical experience with these values is limited, we evaluated their response to bolus administrations of phenylephrine or ephedrine. The relationships between these vasopressors and the additional hemodynamic variables provided by the HPI software were also observed. Both phenylephrine and ephedrine increased MAP, CO and dP/dt, while neither had any significant effect on Ea_dyn_. Both drugs decreased PPV and SVV.

The use of rate of pressure change (dP/dt) measurements to characterize myocardial contractility initially tracked left ventricular (LV) pressure during isovolumetric contraction [13]. This measurement requires direct LV catheterization. Studies which administered assorted inotropes demonstrated dP/dt is very sensitive to changes in contractility in isolated tissues, animals and humans [16]. Further, dP/dt was shown to be more accurate when trending cardiac contractility changes within individual patients in response to acute interventions as opposed to a comparative measure for assessing absolute values of contractility among patients [17]. To expand the clinical utility of this measurement, dP/dt derived from the arterial pressure has been shown to correlate with both direct LV pressure measurements [17, 19] and echocardiographic surrogates [18]. Although the absolute values differ between LV and arterial dP/dt, the response to inotropes and vasodilators within a given patient correlate [17]. The HPI Acumen® software makes peripheral arterial dP/dt continuously available. Characterizing the impact of commonly used vasopressors on this variable will improve the use of this LV contractility measure to guide patient care. However, the limitations of radial arterial pressure measurement in comparison to direct LV pressure measurement should be emphasized, given the multiple potential confounding influences on the radial pressure tracing.

Clinical strategies for resuscitating hypotensive patients rely on understanding fluid responsiveness and vasomotor tone [20]. The ratio of pulse pressure (PP) to stroke volume (SV) was proposed as a reliable estimate of total arterial compliance based on the Windkessel model, which integrates LV SV and the stiffness of an arterial vessel to predict change in arterial pressure [21, 22]. If arterial elastance is held constant, PP is directly proportional to SV, and greater PPV positively correlates with increases in CO following intravenous fluid administration [23]. Expansion upon this model reveals Ea_dyn_, the ratio of PPV to SVV, as a characterization of vasomotor tone. Studies have focused on this parameter’s utility for predicting changes in MAP among patients in the intensive care setting in the response to fluid boluses or vasopressors [7, 24]. Garcia et al. demonstrated that Ea_dyn_ was sufficient to predict arterial pressure change in a study of hypotensive patients under controlled mechanical ventilation after receiving fluids, while neither the ratio PP/SV nor systemic vascular resistance (SVR) predicted a pressure response [22]. Ea_dyn_ assessed arterial tone in resuscitated septic patients was also shown to predict lower MAP after decreased norepinephrine doses were given [24]. Identifying relationships among vasopressors and Ea_dyn_ may strengthen its utility as an indicator of arterial tone perioperatively.

When comparing the effects of the vasopressors on the primary hemodynamic variables for this study, ephedrine demonstrated a greater impact than phenylephrine on MAP, HR, CO and dP/dt. Ephedrine’s greater effect on MAP could be explained by its α_1_-agonist properties which increase SVR in combination with its β-agonist actions and concomitant increase in HR and contractility. In contrast, phenylephrine, as a pure α_1_-agonist, has no corresponding direct effects on HR or contractility. Both drugs were, however, associated with an increase in CO and contractility as assessed by the dP/dt. These observations were not expected for phenylephrine but are consistent with previous observations. The α_1_-mediated vasoconstriction increases both arterial and venous vascular tone. This increase in venous tone may increase left ventricular end-diastolic volume with consequent increased contractility [25]. In animal studies, phenylephrine increased MAP in both preload independent and preload dependent (volume-responsive) states. However, in the volume-responsive state, phenylephrine increased venous return and increased cardiac output [26]. Human studies in clinical settings have not been as consistent. Rebet et al. found that in general surgical patients, phenylephrine consistently increased MAP but decreased CO in patients who were preload independent and had no effect on CO in patients who were preload dependent [27]. In contrast, Kalmar et al. studied the effects of phenylephrine for the treatment of hypotension in patients undergoing elective sigmoidectomy [28]. Nearly all patients were volume-responsive with an average baseline SVV of 19% and CO increased by an average of 26% following administration of phenylephrine [28]. In addition, the increased arterial tone and associated increased afterload might also increase the dP/dt, independent of a change in contractility [13, 29]. Ephedrine will produce all these same actions and, in addition, increase contractility through β_1_-agonist effects resulting in the observed greater effects on HR, CO and contractility as measured by dP/dt.

In contrast, neither phenylephrine nor ephedrine had a significant effect on Ea_dyn_. Ea_dyn_= PPV/SVV. Both drugs decreased SVV and PPV by a similar magnitude. If Ea_dyn_ was a simple measure of vascular tone, vasoconstriction should increase Ea_dyn_. The most common measure of vascular tone, systemic vascular resistance (SVR), calculates the average resistance to blood flow over time that is primarily a function of arteriolar tone. In contrast, Ea_dyn_ uses the dynamic variables of SVV and PPV to provide a more comprehensive characterization of vascular impedance by incorporating the oscillatory nature of blood flow, arterial compliance and blood pressure wave propagation and reflection [22]. Our results emphasize that Ea_dyn_ is a more nuanced value that may predict the blood pressure response to a fluid bolus but is not a simple measure of systemic vascular resistance. For this study, conclusions regarding the comparisons of these two variables are limited because central venous pressure measurements were not available for the calculation of SVR in all patients.

The generalizability of our findings to other surgical populations may be limited since the etiology of intraoperative hypotension is often multifactorial. The efficacy of either dP/dt or Ea_dyn_ as therapeutic guides to treat intraoperative hypotension needs further exploration. We retrospectively analyzed hemodynamic parameters after vasopressor administration in surgeries conducted under general anesthesia. To strengthen the study design and more completely characterize the effects, additional information including the amount of concurrent fluid given to a patient during the hypotensive episode, ventilation mode, and primary anesthetic could be standardized. Prospective, blinded vasopressor treatments would provide more robust comparisons. In addition, in this study patient population of cardiac and general surgery patients, their variable comorbidities, physiologic hemostasis, and susceptibility to hypotension were not controlled.

## Conclusions

To our best knowledge, this study is the first to specifically measure the responses of dP/dt and Ea_dyn_ to vasopressor boluses. Our understanding of how dP/dt and Ea_dyn_ respond to phenylephrine and ephedrine is quantifiably characterized. dP/dt demonstrates potential for characterizing the inotropic response after a bolus of phenylephrine or ephedrine while Ea_dyn_ does not appear to be affected by bolus administration of phenylephrine or ephedrine.

## Data Availability

The datasets used and/or analyzed during the current study are available from the corresponding author on reasonable request.

## List of abbreviaitons

CO: Cardiac Output
dP/dt: Systolic Slope
Ea_dyn_: Dynamic arterial elastance
HPI: Hypotension Prediction Index
HR: Heart rate
IRB: Institutional Review Board variation
LV: Left Ventricular
MAP: Mean Arterial Pressure
PP: Pulse Pressure
PPV: Pulse Pressure Variation
SVR: Systemic Vascular Resistance
SV: Stroke Volume
SSV: Stroke Volume Variation
